# Factors influencing recurrence after in-hospital treatment of odontogenic-related oromaxillofacial infections: a single-centre experience

**DOI:** 10.1186/s12903-025-06662-3

**Published:** 2025-07-28

**Authors:** Andreas Sakkas, Majeed Rana, Mario Scheurer, Robin Kasper, Marcel Ebeling, Frank Wilde, Alisa Schramm, Jasmin Engelbrecht

**Affiliations:** 1 Department of Oral and Maxillofacial Surgery, University Hospital Ulm, Albert-Einstein-Allee 11, 89081 Ulm, Germany; 2 Department of Oral and Plastic Maxillofacial Surgery, Military Hospital Ulm, Ulm, Germany; 3 Department of Otolaryngology, Head and Neck Surgery, Military Hospital Ulm, Ulm, Germany

**Keywords:** Oromaxillofacial infection, Odontogenic abscess, In-hospital treatment, Recurrence

## Abstract

**Purpose:**

Evidence regarding specific factors influencing recurrence after treatment of oromaxillofacial infections is limited. This study aimed to evaluate the incidence and identify predictors of recurrence following in-hospital treatment. A secondary aim was to analyze associations between patient- and procedure-specific variables, treatment outcomes, and hospital length of stay (LOS), and to identify high-risk patients.

**Methods:**

In this retrospective, single-center study, patients with odontogenic-related oromaxillofacial infections treated surgically or conservatively over a 4-year period were included. Demographic, clinical, radiological, and treatment data were analyzed. Multivariable analyses were performed to identify predictors of recurrence and LOS.

**Results:**

A total of 939 patients (mean age 44.66 ± 22.95 years) met the inclusion criteria. The recurrence rate was 5.01%, and the mean LOS was 3.55 ± 2.47 days. Increased age and BMI, mandibular infections, infections post-extraction or post-augmentation, clinical symptoms at admission (restricted mouth opening, dysphagia, non-palpable mandibular margin), elevated inflammatory markers (CRP, leukocytes, neutrophils, procalcitonin), and cervical drainage were significantly associated with higher recurrence risk and increased LOS (*p* < 0.05). A postoperative ICU stay increased the recurrence risk 9.13-fold.

**Conclusions:**

Within the limitations of this study, the results suggest that older age, higher BMI, mandibular involvement, post-extraction or post-augmentation infections, specific clinical symptoms at admission (restricted mouth opening, dysphagia, non-palpable mandibular margin), elevated inflammatory markers, cervical drainage approaches, and stay in ICU are associated with both a higher risk of infection recurrence and increased hospital stay. Multiple bacterial strains and no continuous antibiotic therapy after discharge also indicated a higher risk of recurrence, while surgery under intubation anesthesia was linked to an increased length of stay only. These factors should be considered early in treatment planning to optimize outcomes.

**Clinical trial number:**

Not applicable.

## Introduction

Despite improvements in oral health, broader access to dental care, and the widespread use of broad-spectrum antibiotics, infections of the oral and maxillofacial region remain a significant burden on public health systems [1–8]. Several studies have reported a rise in emergency department admissions for these infections, contributing to increasing healthcare costs [1–3, 8].

Oromaxillofacial infections may arise from various odontogenic pathologies, including chronic apical periodontitis, dental caries, and pericoronitis, as well as complications following dental procedures such as implant placement, bone grafting, or osteomyelitis of different origins with or without prodromic symptoms, with and without preoperative medications and operations to reduce the incidence [3, 5, 9–14]. Microbiological studies revealed mixed bacterial flora, predominantly composed of oral commensals with variations based on the specific oral site [11, 15–17].

Treatment of infections in this anatomically complex region is challenging. Factors such as bacterial virulence, impaired host immunity, and inadequate treatment can allow infections to spread to deep spaces of the head and neck, resulting in severe complications, prolonged hospitalization, ICU admissions, and even mortality [1–7, 9–11]. Rapid diagnosis and appropriate therapy are critical for favorable outcomes, reduced hospitalization times, and prevention of systemic complications, as emphasized in previous studies [9–11].

Diagnostic workup should include comprehensive clinical and radiological evaluations to support differential diagnosis [7, 11]. Therapeutic management depends on various factors, including the source and severity of infection. Key components include airway management, empiric or targeted antibiotic therapy, surgical drainage, removal of the infection source, and appropriate dental treatment [5, 9–11, 18, 19].

Although the need for empiric antibiotic therapy has been debated, it remains necessary until culture-based antimicrobial guidance is available [7, 11]. However, no consensus exists regarding standardized treatment protocols concerning surgical intervention, the type and duration of antibiotic therapy, or criteria for hospitalization [7, 19].

Even after seemingly successful therapy, oromaxillofacial infections may recur. Recurrence may be influenced by patient-related factors (e.g., smoking, immunosuppression, diabetes, antiresorptive therapy), poor oral hygiene, and patient non-compliance. Microbiological factors, including polymicrobial infections and bacterial resistance, can also impair therapeutic success. Furthermore, treatment-related factors such as insufficient antibiotic duration, delayed surgical intervention, inadequate debridement, or residual infection foci can contribute to recurrence.

At our center, odontogenic or iatrogenic infections constitute a frequent cause of hospitalization. Despite advances in diagnostic and therapeutic approaches, infection recurrence remains a significant clinical challenge. However, the specific factors contributing to persistence or recurrence have not been clearly identified in the existing literature.

Understanding the interplay between patient-related, microbiological, and treatment-related factors is critical for preventing recurrence. Clinicians must consider these aspects during treatment planning and educate patients on the importance of oral hygiene and treatment adherence. A comprehensive approach can optimize outcomes and minimize recurrence risk.

The primary aim of this study was to evaluate the incidence and identify factors associated with recurrence of oromaxillofacial infections following in-hospital treatment. The secondary aim was to assess the association between patient- and treatment-specific variables with hospital length of stay (LOS) and to identify high-risk patients.

## Methods

### Patient cohort

This observational, retrospective single-center study included patients with odontogenic-related oromaxillofacial infections who received either surgical or conservative in-hospital treatment at the Department of Oral and Maxillofacial Surgery between September 2019 and April 2023. Medical records were retrieved from the hospital’s electronic database. Ethical approval was obtained from the Ethics Committee of the Chamber of Physicians in Baden-Württemberg, Ulm, Germany (approval number: 189/23; approval date: 20 July 2023). The study was conducted in accordance with the Declaration of Helsinki (1964) and its subsequent amendments.

Inclusion criteria were: (1) patients of any age, (2) clinically and radiologically confirmed odontogenic-related infections originating from the maxilla or mandible, (3) patients treated surgically (with antibiotics) or conservatively (with antibiotics only), and (4) hospitalization following diagnosis and treatment decision. Exclusion criteria were: (1) non-odontogenic orofacial infections, (2) outpatient treatment only, and (3) incomplete medical records.

### Patient screening

Diagnosis of odontogenic-related infections followed a standardized protocol including clinical assessment, laboratory testing, and radiological imaging. Radiological evaluation involved panoramic radiographs and/or computed tomography (CT) scans to assess the infection source, extent, and airway involvement. Clinical assessment focused on swelling, dysphagia, dyspnea, stridor, palpability of the mandibular margin, and mouth opening. Hospitalization and treatment decisions (surgical vs. conservative) were made by board-certified oral and maxillofacial surgeons.

Odontogenic sources included chronic apical periodontitis, dental caries, odontogenic and non-odontogenic cysts, pericoronitis, peri-implantitis, and complications following dental extraction, implant placement, or bone augmentation surgery.

### Infection treatment

Antibiotic therapy was initiated at admission and continued until discharge for both surgical and conservatively treated patients. Standard antibiotic regimens included ampicillin-sulbactam (Unacid©) at 3 g three times daily for patients > 70 kg and 2 g three times daily for patients ≤ 70 kg. Pediatric doses were adjusted to 150 mg/kg/day. Patients allergic to penicillin received clindamycin (600 mg three times daily).

Abscess incision was performed intraorally or extraorally under local or general anesthesia, depending on the origin, location, size of the abscess, and the patient’s clinical condition. For extraoral drainage, a submandibular skin-fold incision was made, preserving the marginal branch of the facial nerve. Drainage tubes were inserted following debridement. Microbiological samples were obtained after pus drainage, and wounds were irrigated with disinfectant solutions.

The dental source of the infection, which was considered to have a poor prognosis, was removed either during the same procedure or in a follow-up intervention, depending on clinical severity. This could occur either during hospitalization or at an outpatient follow-up. If the prognosis was positive, the patient was referred to our outpatient clinic or to the referring dental practitioner after completing of the initial in-hospital treatment in order to initiate root canal treatment.

### Data collection

Patient data were extracted from electronic hospital records and anonymized prior to analysis. Data collected included: age, gender, body mass index (BMI), smoking status, diabetes mellitus, arterial hypertension, penicillin allergy, laboratory results, infection localization, clinical symptoms at admission (mouth opening, subjective odynophagia, extraoral mandibular margin palpability), infection etiology, treatment method (surgical/conservative), surgical access (intraoral/extraoral), antibiotic therapy details, anesthesia type (local anesthesia, intubation anesthesia, sedation, laryngeal mask), microbiological data, and outcomes (ICU stay, length of stay, recurrence, duration till recurrence).

**Fig. 1:**
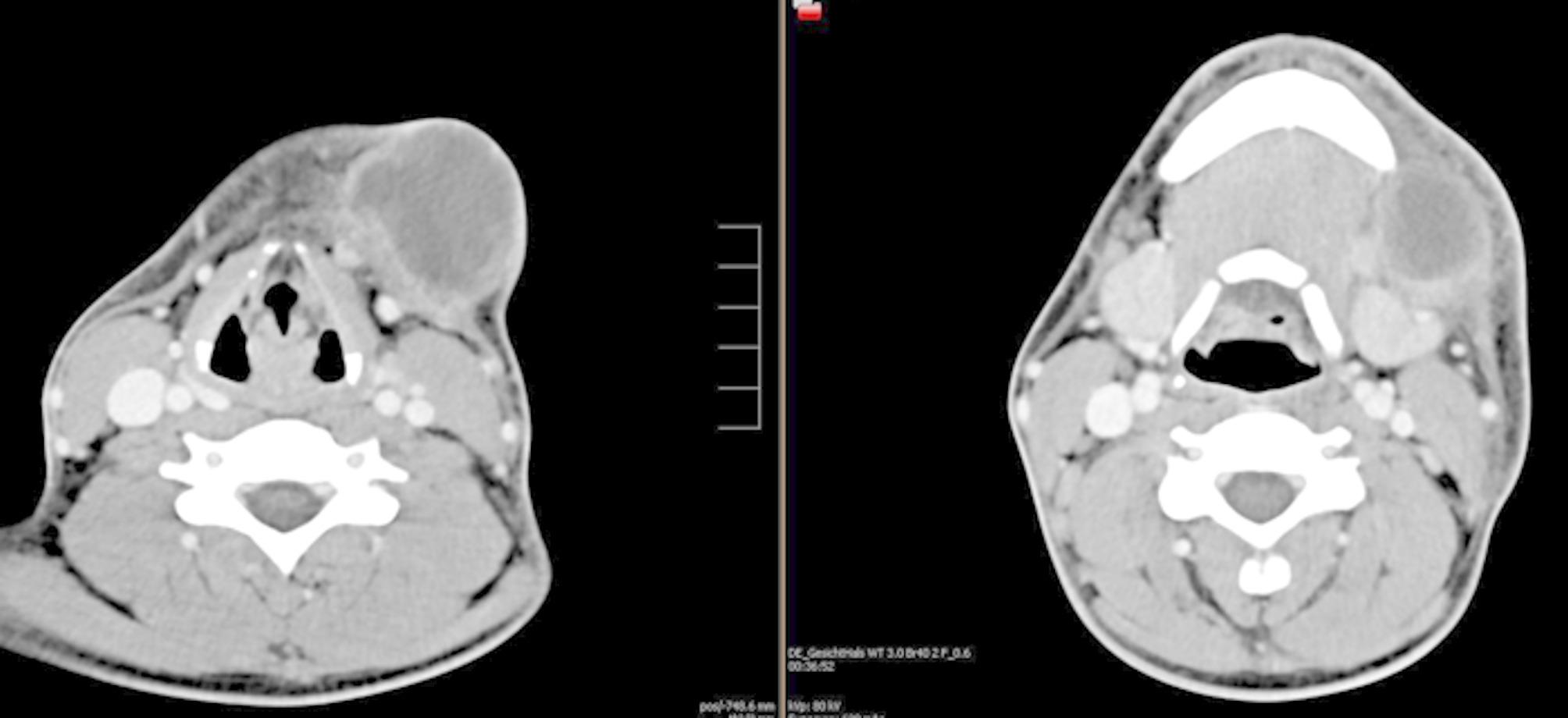
Axial contrast-enhanced computed tomography (CT) scan demonstrating a perimandibular abscess on the left side. The image shows a hypodense, rim-enhancing fluid collection adjacent to the left mandibular body, with surrounding soft tissue swelling and signs of inflammatory infiltration

**Fig. 2:**
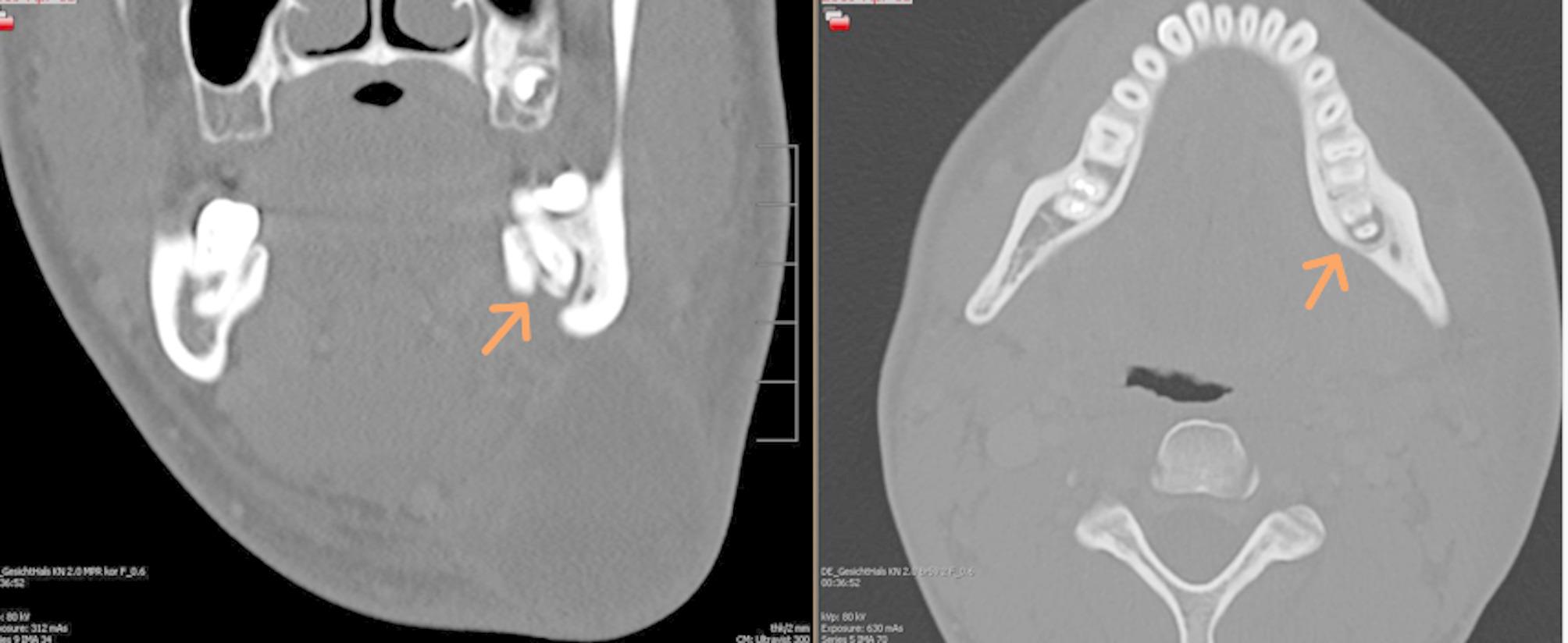
Coronal and axial contrast-enhanced computed tomography (CT) images illustrating the odontogenic origin of the infection. The scans reveal an apical osteolytic lesion associated with the left second mandibular molar, indicating it as the causative focus for the development of the perimandibular abscess

Radiological findings relevant to abscess formation in orofacial spaces (e.g., paramandibular, submandibular, perimandibular, mouth floor, submental, masseteric, pterygomandibular, parapharyngeal) were documented. Two board-certified radiologists independently reviewed all imaging for interrater reliability (Figs. 1 and 2).

### Follow-up

Clinical follow-up assessments were performed to confirm wound healing, resolution of symptoms, and absence of infection recurrence.

Recurrence was defined as the reappearance of infection-related symptoms (e.g., soft tissue swelling, purulent discharge, persistent pain) in the same anatomical location as the initial infection, with radiological evidence present within a follow-up period of 6 months after completion of the initial in-hospital treatment. All patients showing clinical deterioration suggestive of infection recurrence were readmitted for additional in-hospital treatment. Repeat imaging was performed only when recurrence was clinically suspected.

Since post-hospital follow-up care was provided by various referring dental practitioners or maxillofacial surgeons, patients who did not return to our clinic within 6 months after the initial in-hospital treatment and from whom no complications were reported were presumed to have healed successfully. Therefore, aside from the recurrence cohort, it was not possible to collect information on whether the infection source identified during the initial infection in cases with a good prognosis was treated or not after the initial in-hospital treatment. The follow-up period was only documented in the recurrence cohort, which represents the time between the initial in-hospital treatment and the readmission following a recurrence diagnosis.

### Study variables

Potential predictor variables included:

- **Demographic**: Age (< 30, 30–49, ≥ 50 years), gender.
- **Anamnestic**: BMI (< 25, 25–29.9, ≥ 30), smoking status, diabetes mellitus, arterial hypertension, penicillin allergy.
- **Clinical**: Etiology, location, jaw involved, clinical symptoms (restricted mouth opening, dysphagia, mandibular margin palpability).
- **Laboratory**: CRP (mg/dL), WBC (/nL), absolute neutrophil count (%), procalcitonin (ng/mL).
- **Microbiology**: Number and type of bacterial strains (aerobic/anaerobic).
- **Treatment**: Treatment method, surgical approach (intraoral/extraoral), antibiotic regimen, ICU admission, hospital length of stay.

Primary outcome: infection recurrence.

Secondary outcome: hospital length of stay (LOS). LOS greater than 5 days (≥ 5) was defined as prolonged.

### Statistical analysis

Data were compiled electronically using Microsoft Excel and analyzed with SAS^®^, Release 9.4 (SAS Institute Inc., Cary, NC, USA). Descriptive statistics were used to summarize baseline characteristics. Categorical variables were expressed as counts and percentages.

The Kolmogorov-Smirnov test confirmed non-normal distribution for age and LOS; thus, LOS and follow-up were reported as medians with ranges. Associations between categorical variables and outcomes were tested using chi-square or Fisher’s exact tests. Multivariable logistic regression models were performed to identify independent predictors for infection recurrence. Predictor variables were selected based on clinical relevance and univariate significance (*p* < 0.1), while considering the limited number of recurrence events (*n* = 47) to minimize the risk of overfitting. Variables with excessive missing data (e.g., BMI and PCT) were excluded from the multivariable analysis to minimize bias. The number of predictors included in the model was limited in accordance with the number of observed recurrence events to reduce the risk of overfitting. Spearman correlations and chi-square tests analyzed associations with LOS. Dichotomization of LOS using a 5-day threshold for statistical analysis was avoided, as this cutoff value is based primarily on clinical experience rather than evidence-based outcome data. To preserve the full variability and potential prognostic value of this parameter, LOS was retained as a continuous variable in the analysis. A p-value ≤ 0.05 was considered statistically significant.

## Results

### Demographic distribution

A total of 939 patients met the inclusion criteria. The cohort consisted of 532 males (56.6%) and 407 females (43.3%), yielding a male-to-female ratio of 1.30:1. The mean age at admission was 44.66 ± 22.95 years, ranging from 0 to 95 years. Patients aged ≥ 50 years represented the largest subgroup (*n* = 393; 41.8%). The most frequent age group among males (*n* = 196; 36.8%) and females (*n* = 197; 48.4%) was ≥ 50 years. A total of 246 patients (26.20%) reported being active smokers. The distribution of demographic, anamnestic, microbiological, and treatment-related predictor variables in relation to infection recurrence is summarized in Table 1.

**Table 1:** Distribution of predictor variables in correlation with infection recurrence

		No infection recurrence		Infection recurrence					
Predictor variables									*p* value
		n	% of variable group	n	% of variable group	% of recurrence	Total	% of the sample	
*Demographic variables*									
**Age at admission**									0.093*
< 30 years		257	96.25%	10	3.75%	21.28%	267	28.43%	
30–50 years		268	96.06%	11	3.94%	23.40%	279	29.71%	
≥ 50 years		367	93.38%	26	6.62%	55.32%	393	41.85%	
**Gender**									
male		510	95.86%	22	4.14%	46.81%	532	56.66%	0.162**
female		382	93.86%	25	6.14%	53.19%	407	43.34%	
*Anamnestic variables*									
**BMI**									0.126*
< 25		181	93.30%	13	6.70%	27.65%	194	20.66%	
25–30		80	87.91%	11	12.09%	23.40%	91	9.69%	
≥ 30		57	87.69%	8	12.31%	17.02%	65	6.92%	
NA		574	97.45%	15	2.54%	31.91%	589	62.73%	
**Smoking status**									0.530**
Yes		232	94.31%	14	5.69%	29.79%	246	26.20%	
No		652	95.32%	32	4.68%	68.09%	684	72.84%	
suspended		8	88.89%	1	11.11%	2.13%	9	0.96%	
**Diabetes mellitus**									0.155**
Yes		93	92.08%	8	7.92%	17.02%	101	10.76%	
No		799	95.35%	39	4.65%	82.98%	838	89.24%	
**Arterial hypertension**									0.366**
Yes		214	82.98%	14	6.14%	29.79%	228	24.28%	
No		678	95.36%	33	4.64%	70.21%	711	75.72%	
**Penicillin allergy**									0.454***
Yes		38	92.68%	3	7.32%	6.38%	41	4.37%	
No		854	95.10%	44	4.90%	93.62%	898	95.63%	
*Microbiology variables*									
**Bacteria culture**									0.053**
aerobic		108	91.53%	10	8.47%	28.57%	118	18.82%	
anaerobic		173	94.02%	11	5.98%	31.43%	184	29.35%	
both		101	91.82%	9	8.18%	25.71%	110	17.54%	
normal flora		210	97.67%	5	2.33%	14.29%	215	34.29%	
*Treatment variables*									
**Treatment method**									0.950***
Surgical intervention + antibiotics		833	94.98%	44	5.02%	93.62%	877	93.40%	
Antibiotics only		59	95.16%	3	6.38%	4.84%	62	6.60%	
**Surgical approach für drainage**									0.001***
cervical		58	85.29%	10	14.71%	22.73%	68	7.75%	
intraoral		719	96.12%	29	3.88%	65.91%	748	85.29%	
both		56	91.80%	5	8.20%	11.36%	61	6.96%	
**Preoperative antibiotic therapy**									0.480**
Yes		231	95.85%	10	4.15%	21.28%	241	25.67%	
No		661	94.70%	37	5.30%	78.72%	698	74.33%	
**Antibiotic therapy after discharge**									0.025**
Yes		223	97.81%	5	2.19%	10.64%	228	24.28%	
No		669	94.09%	42	5.91%	89.36%	711	75.72%	
**Postoperative stay in ICU**									0.003***
Yes		9	69.23%	4	30.77%	8.51%	13	1.38%	
No		883	95.36%	43	4.64%	91.49%	926	98.62%	

Abbreviations: n=Number; %=Percentage; NA=Not applicable; ICU=Intensive Care Unit

* Mann-Whitney U-Test; ** Chi²-Test; *** Fisher’s exact Test

^1^related to 627 patients with microbiology test

### Etiology and location

Infections were located in the mandible in 66.67% of cases (*n* = 626) and in the maxilla in 33.33% (*n* = 313). Chronic apical periodontitis was the leading cause (58.15%; *n* = 546), followed by post-extraction infections (18.10%; *n* = 170) and dental caries (16.19%; *n* = 152).

The most frequently involved anatomical spaces were the paramandibular space (*n* = 294; 31.31%), canine fossa (*n* = 180; 19.17%), and perimandibular space (*n* = 118; 12.57%). The infection most commonly affected the first molar region (*n* = 188; 20.02%), followed by the third molar (*n* = 160; 17.04%) and the anterior teeth region (*n* = 155; 16.51%). Table 2 presents the distribution of clinical variables, including etiology and infection site characteristics, in correlation with infection recurrence.

**Table 2:** Distribution of clinical variables in correlation with infection recurrence

		No infection recurrence		Infection recurrence				
**Clinical variables**							Total	**p value**
		n	% of variable group	n	% of variable group	% of recurrence		
**Etiology of infection**								
Chronic apical parodontitis		520	95.24%	26	4.76%	55.32%	546	0.687*
Post-extraction		155	91.18%	15	8.82%	31.91%	170	0.012*
Caries		151	99.34%	1	0.66%	2.13%	152	0.056*
Pericoronitis		24	100%	0	0%	0%	24	0.627**
Parodontitis		17	100%	0	0%	0%	17	1.000**
Post implantation		4	66.67%	2	33.33%	4.26%	6	0.032**
Post augmentation		3	60%	2	40%	4.26%	5	0.022**
Post anterior tooth trauma		5	100%	0	0%	0%	5	1.000**
Post explantation		3	100%	0	0%	0%	3	1.000**
Post apicectomy		2	100%	0	0%	0%	2	1.000**
Unsuccessful extraction attempt		2	100%	0	0%	0%	2	1.000**
Tooth preparation trauma		0	0%	1	100%	2.13%	1	0.050**
Globulomaxillary cyst		1	100%	0	0%	0%	1	1.000**
follicular cyst		1	100%	0	0%	0%	1	1.000**
Post implant exposure		1	100%	0	0%	0%	1	1.000**
Spontaneous implant failure		1	100%	0	0%	0%	1	1.000**
Periimplantitis		1	100%	0	0%	0%	1	1.000**
Infected gingival cyst		1	100%	0	0%	0%	1	1.000**
**Location of infection**								
Paramandibular		284	96.6%	10	3.4%	21.28%	294	0.128*
Canine fossa		176	97.78%	4	2.22%	8.51%	180	0.057*
Perimandibular		108	91.53%	10	8.47%	21.28%	118	0.065*
Submucous		61	100%	0	0%	0%	61	0.067**
Submandibular		52	89.66%	6	10.34%	12.77%	58	0.063**
Paramaxillary posterior		43	100%	0	0%	0%	43	0.162**
Posterior floor of the mouth		38	92.68%	3	7.32%	6.38%	41	0.454**
Pterygomandibular		32	91.43%	3	8.57%	6.38%	35	0.413**
Buccal		32	91.43%	3	8.57%	6.38%	35	0.413**
Palatal		15	100%	0	0%	0%	15	1.000**
Anterior floor of the mouth		10	71.43%	4	28.57%	8.51%	14	0.056**
Paramaxillary anterior		14	100%	0	0%	0%	14	1.000**
Submental		11	84.62%	2	15.38%	4.26%	13	0.135**
Mental		8	100%	0	0%	0%	8	1.000**
Periodontal		4	100%	0	0%	0%	4	1.000**
Parapharyngeal		2	66.67%	1	33.33%	2.13%	3	0.143**
Retromaxillary		2	66.67%	1	33.33%	2.13%	3	0.143**
**Affected jaw**								0.002*
Maxilla		307	98.08%	6	1.92%	12.77%	313	
Mandible		585	93.45%	41	6.55%	87.23%	626	
**Mouth opening**								0.003*
Normal		490	96.84%	16	3.16%	34.04%	506	
Restricted		385	92.55%	31	7.45%	65.96%	416	
NA							17	
**Subjective odynophagia**								**0.001**
Yes		178	90.36%	19	9.64%	40.43%	197	
No		714	96.23%	28	3.77%	59.57%	742	
**Extraoral palpability of the mandibular basal margin**								**0.018**
Palpable		435	95.19%	22	4.81%	53.66%	457	
Non-palpable		143	89.94%	16	10.06%	39.02%	158	
NA							324	

Abbreviations: n = number; %=percentage; NA = not applicable; ICU = Intensive Care Unit

* Chi²-Test; ** Fisher’s exact Test

### Clinical symptoms and laboratory findings

Upon admission, restricted mouth opening was observed in 416 patients (44.30%). Dysphagia was reported by 197 patients (20.98%), and dyspnea by 11 patients (1.17%).

Among patients with mandibular infections (*n* = 626), the mandibular basal margin was non-palpable in 159 cases (25.40%).

Body temperature at admission was recorded in 720 patients, with a mean of 36.85 ± 0.66 °C (range: 35.0–40.0 °C). Normothermia (36.3–37.4 °C) was observed in 523 cases (55.70%). Associations between continuous numeric variables and infection recurrence were further assessed using linear regression models, as shown in Table 3.

**Table 3:** Linear regression analysis of numeric predictor variables and infection recurrence

Infection recurrence						
	*n*	mean	SD	Minimum	Maximum	*p* value
	**Age**					0.009*
No	892	44.21	22.89	0	95	
Yes	47	53.17	22.9	10	92	
	**BMI** ^**¤**^					0.031*
No	318	23.95	6.78	10.5	46.28	
Yes	32	26.64	5.62	15.79	40.15	
	**Temperature °C** ^**¤**^					0.889**
No	686	36.85	0.66	35	40	
Yes	34	36.9	0.78	35.8	39.2	
	**CRP (mg/dl)** ^**¤**^					0.004**
No	819	6.26	6.44	0.06	37.4	
Yes	46	10.4	9.75	0.2	34.61	
	**WBC count (/nL)** ^**¤**^					0.042**
No	818	11.790	4.370	2.100	56.100	
Yes	47	13.230	5.230	6.100	33.700	
	**ANC count (%)** ^**¤**^					< 0.001**
No	811	72.46	9.08	14	95	
Yes	47	77.34	8.65	49	96	
	**PCT level (ng/ml)** ^**¤**^					0.022**
No	52	1.59	6.39	0.03	40.42	
Yes	9	3.64	8.99	0.11	27.49	
	**Number of bacterial strains** ^**¤**^					0.022**
No	592	1.11	1.1	0	5	
Yes	35	1.51	1.04	0	4	

Abbreviations: n = number; %=percentage; SD = Standard Deviation; ICU = Intensive Care Unit; BMI = Body Mass Index; CRP = C-Reactive protein; WBC = White Blood Cells; ANC = Absolut Neutrophil Count; PCT = Procalcitonin

* t-Test; ** Mann-Whitney U-Test

^**¤**^ relating only to the documented case number

### Infection treatment and length of stay

Surgical intervention was performed in 877 patients (93.40%), while 62 patients (6.60%) received conservative antibiotic treatment only. The majority (*n* = 748; 85.29%) underwent intraoral incision and drainage; 68 patients (7.75%) underwent cervical/submandibular drainage, and 61 patients (6.96%) required both approaches.

Pre-admission antibiotic therapy had been administered to 241 patients (25.67%), most commonly with ampicillin-sulbactam (*n* = 810; 92.36%) or clindamycin (*n* = 50; 5.70%). Postoperative antibiotic regimens were similar, with ampicillin-sulbactam prescribed in 804 cases (91.68%) and clindamycin in 48 cases (5.47%). Predictor variables associated with the length of hospital stay are summarized in Table 4.

**Table 4:** Distribution of predictor variables in correlation with length of stay

Length of stay						
	*n*	mean	SD	Minimum	Maximum	*p* value
*Demographic variables*						
**Age at admission**						< 0.001*
< 30 years	267	2.82	1.87	0	12	
30–50 years	279	3.57	1.9	1	18	
≥ 50 years	393	4.03	3	0	47	
**Gender**						0.863**
Male	532	3.54	2.64	0	47	
Female	407	3.55	2.22	0	18	
*Anamnestic variables*						
**BMI**						< 0.001*
< 25	194	3.35	2.59	0	12	
25–30	91	5.51	5.09	1	47	
≥ 30	65	5.14	2.75	1	18	
**Smoking status**						0.154**
Yes	246	3.64	1.78	1	11	
No	684	3.51	2.68	0	47	
suspended	9	3.67	1.94	1	6	
**Diabetes mellitus**						0.059**
Yes	101	3.89	2.25	0	14	
No	838	3.5	2.49	0	47	
**Arterial hypertension**						0.001**
Yes	228	4.01	3.47	1	47	
No	711	3.4	2.02	0	18	
**Penicillin allergy**						0.701**
Yes	41	3.61	1.91	1	12	
No	898	3.54	2.49	0	47	
*Microbiology variables*						
**Bacteria culture**						0.451*
Aerobic	118	4.15	2.66	1	18	
Anaerobic	184	3.82	2.05	1	12	
Both	110	4.25	4.56	1	47	
Normal flora	215	3.63	1.94	0	14	
*Treatment variables*						
**Method of anesthesia**						< 0.001**
Local anesthesia	554	3.27	1.52	0	18	
Intubation anesthesia	266	4.74	3.65	0	47	
Sedation	36	1.17	0.61	0	4	
Laryngeal mask	21	1.38	1.43	0	7	
No anesthesia	62	3.05	1.26	1	5	
**Treatment method**						
Surgical intervention + antibiotics	877	3.58	2.53	0	47	0.193**
Antibiotics only	62	3.05	1.26	1	5	
**Surgical approach für drainage**						
Cervical	68	5.96	5.41	0	47	
Intraoral	748	3.17	1.77	0	18	
Both	61	6.03	2.59	1	18	
**Preoperative antibiotic therapy**						0.222***
Yes	241	3.71	2.09	0	18	
No	698	3.49	2.58	0	47	
**Antibiotic therapy after discharge**						0.013***
Yes	228	2.09	1.29	1	12	
No	711	4.01	2.57	0	47	
**Postoperative stay in ICU**						0.028**
Yes	13	11.23	11.21	5	47	
No	926	3.44	1.92	0	18	

Abbreviations: n = number; %=percentage; ICU = Intensive Care Unit; SD = Standard Deviation

* Kruskal-Wallis test; ** Mann-Whitney U-test; *** t- test

The mean hospital length of stay (LOS) was 3.55 ± 2.47 days (range: 0–47 days). A prolonged LOS (≥ 5 days) was reported in 227 (24.17%) patients. Thirteen patients (1.38%) required postoperative ICU care, with an average ICU stay of 3.92 ± 5.54 days (range: 1–21 days). The results of the linear regression analysis of clinical variables influencing hospital length of stay are presented in Table 5.

**Table 5:** Linear regression analysis of clinical variables and length of stay

Length of stay						
	*n*	mean	SD	Minimum	Maximum	*p* value
**Clinical variables**						
**Etiology of infection**						
Chronic apical parodontitis	546	3.54	2.64	1	47	0.617*
Post-extraction	170	4.28	2.05	0	12	< 0.001*
Caries	152	2.66	1.87	0	11	< 0.001*
Pericoronitis	24	3.71	1.4	1	7	0.253*
Parodontitis	17	3.29	1.76	1	6	0.870*
Post implantation	6	5.83	6.08	2	18	0.403*
Post augmentation	5	5.4	1.67	4	8	0.019*
Post anterior tooth trauma	5	0.6	0.55	0	1	< 0.001*
Post explantation	3	5.67	3.21	2	8	0.192*
Post apicectomy	2	2.5	0.71	2	3	-
Unsuccessful extraction attempt	2	3	0	3	3	-
Tooth preparation trauma	1	5	-	5	5	-
Globulomaxillary cyst	1	4	-	4	4	-
follicular cyst	1	8	-	8	8	-
Post implant exposure	1	3	-	3	3	-
Spontaneous implant failure	1	5	-	5	5	-
Periimplantitis	1	1	-	1	1	-
Infected gingival cyst	1	2	-	2	2	-
**Location of infection**						
Paramandibular	294	3.11	1.6	0	12	< 0.001*
Canine fossa	180	2.72	1.25	0	7	< 0.001*
Perimandibular	118	4.97	2.15	0	14	< 0.001*
Submucous	61	2.33	1.39	0	6	< 0.001*
Submandibular	58	4.48	1.88	1	9	< 0.001*
Paramaxillary posterior	43	2.84	1.46	1	7	0.016*
Posterior floor of the mouth	41	4.46	2.7	1	18	0.004*
Pterygomandibular	35	6.4	7.32	2	47	< 0.001*
Buccal	35	3.77	1.99	1	10	0.323*
Palatal	15	2.67	0.98	1	4	0.096*
Anterior floor of the mouth	14	4.64	4.07	1	18	0.276*
Paramaxillary anterior	14	2.21	1.25	1	5	0.007*
Submental	13	4.69	2.39	1	10	0.038*
Mental	8	2.75	0.71	2	4	0.260*
Periodontal	4	2.5	2.38	1	6	0.218*
Parapharyngeal	3	7.67	2.08	6	10	0.007*
Retromaxillary	3	8.33	3.21	6	12	0.007*
**Affected jaw**						< 0.001*
Maxilla	313	2.78	1.47	0	12	
Mandible	626	3.93	2.76	0	47	
**Mouth opening**						< 0.001*
Normal	506	2.95	1.61	0	11	
Restricted	416	4.36	3.05	0	47	
NA	17	1.35	0.7	0	3	
**Subjective odynophagia**						
Yes	197	4.98	3.9	0	47	< 0.001*
No	742	3.16	1.73	0	12	
**Extraoral palpability of the mandibular basal margin**						< 0.001*
Palpable	457	3.37	1.8	0	18	
Non-palpable	159	5.44	4.07	0	47	
NA	324	-	-	-	-	

Abbreviations: n = number; %=percentage; NA = not applicable; ICU = Intensive Care Unit

* Mann-Whitney U-test

### Infection recurrence

Of the 939 patients, 47 (5.01%) experienced postoperative recurrence. Among them, 28 (59.7%) patients had a prolonged LOS. The most common sites of recurrent swelling were the cervical (19.15%), paramandibular (19.15%), submandibular (17.02%), and buccal regions (12.77%).

In 16 patients (34.04%), the primary odontogenic cause was still present at the time of recurrence diagnosis.

Among the patients with recurrence, 41 (87.23%) underwent surgical re-intervention combined with antibiotic therapy, while six (12.77%) were treated conservatively with antibiotics alone.

The mean time to recurrence was 21.7 ± 30.43 days. Significant predictors for infection recurrence identified through multivariable analysis are summarized in Table 6.

**Table 6:** Significant predictor variables for higher risk of infection recurrence

Significant predictor variables	Odds Ratio	95% CI: x-y	*p* value	Higher risk of infection recurrence
Age	1.02	0.9–1.1	0.009	≥ 50 years
BMI	1.06	0.9–1.2	0.031	≥ 30
Affected jaw	3.59	1.5–8.6	0.002	Mandible
Etiology of infection	2.23	1.0-3.7	0.012	Post-extraction
	9.87	3.5–38.0	0.032	Post-implantation
	13.17	4.5–38.0	0.022	Post-augmentation
Mouth opening	2.47	1.1–5.8	0.003	Restricted
Odynophagia	2.72	1.1–6.2	0.001	Present
Extraoral palpability of the mandibular basal margin	2.21	1.0-4.9	0.018	Non-palpable
CRP level, mg/dl	1.07	0.9–1.2	0.004	↑
WBC count /nL	1.06	0.9–1.2	0.042	↑
ANC count (%)	1.08	0.9–1.3	< 0.001	↑
PCT level, ng/ml	1.03	0.8–1.3	0.022	↑
Surgical approach for drainage	4.33	1.8–10.4	0.001	Cervical
Antibiotic therapy after discharge	2.8	0.1–0.9	0.025	No antibiotic therapy
Multiple bacterial strains (≥ 2)	1.35	0.7–2.6	0.022	↑
ICU stay	9.13	2.8–29.5	0.003	Yes

Abbreviations: CI = Confidence interval; BMI = Body Mass Index; CRP = C-Reactive protein; WBC = White Blood Cells; ANC = Absolut Neutrophil Count; PCT = Procalcitonin; ICU = Intensive Care Unit

### Clinical recurrence risk score

Based on our multivariable analysis, we developed a structured Clinical Recurrence Risk Score to provide clinicians with an actionable tool for the early identification of high-risk patients with odontogenic oromaxillofacial infections.

The score incorporates key predictors significantly associated with infection recurrence, including age, BMI, mandibular infection, post-extraction, post-implantation and post-augmentation infection, trismus, odynophagia, non-palpable mandibular margin, cervical drainage, elevated inflammatory markers (CRP, WBC, ANC, PCT), polymicrobial infections and postoperative ICU stay.

Each risk factor was weighted according to its odds ratio, resulting in a cumulative score ranging from 0 to 31 points. The proposed risk categories are defined as follows: low risk (0–6 points), moderate risk (7–13 points), and high risk (≥ 14 points).

Patients classified as high-risk — especially those with cervical drainage, ICU stay, or infections following implantation or augmentation procedures — may benefit from more aggressive surgical management, longer hospitalization, and intensified follow-up to reduce recurrence rates.

**Fig. 3:**
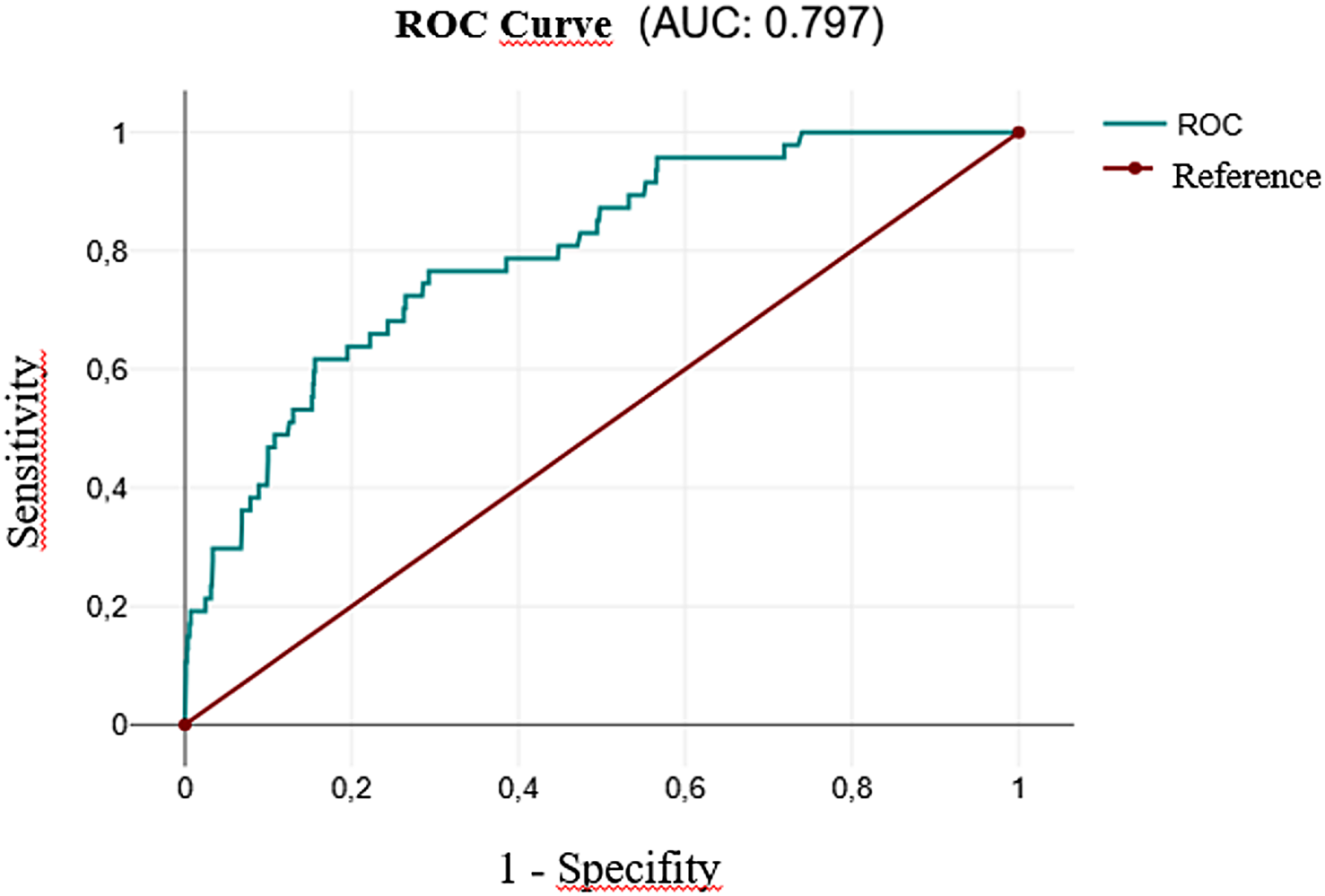
ROC curve showing the discriminatory ability of the Recurrence Risk Score (AUC = 0.797)

The internal validation of the Recurrence Risk Score using receiver operating characteristic (ROC) analysis yielded an area under the curve (AUC) of 0.797, indicating acceptable discriminatory ability. The ROC curve is shown in Fig. 3.

**Fig. 4:**
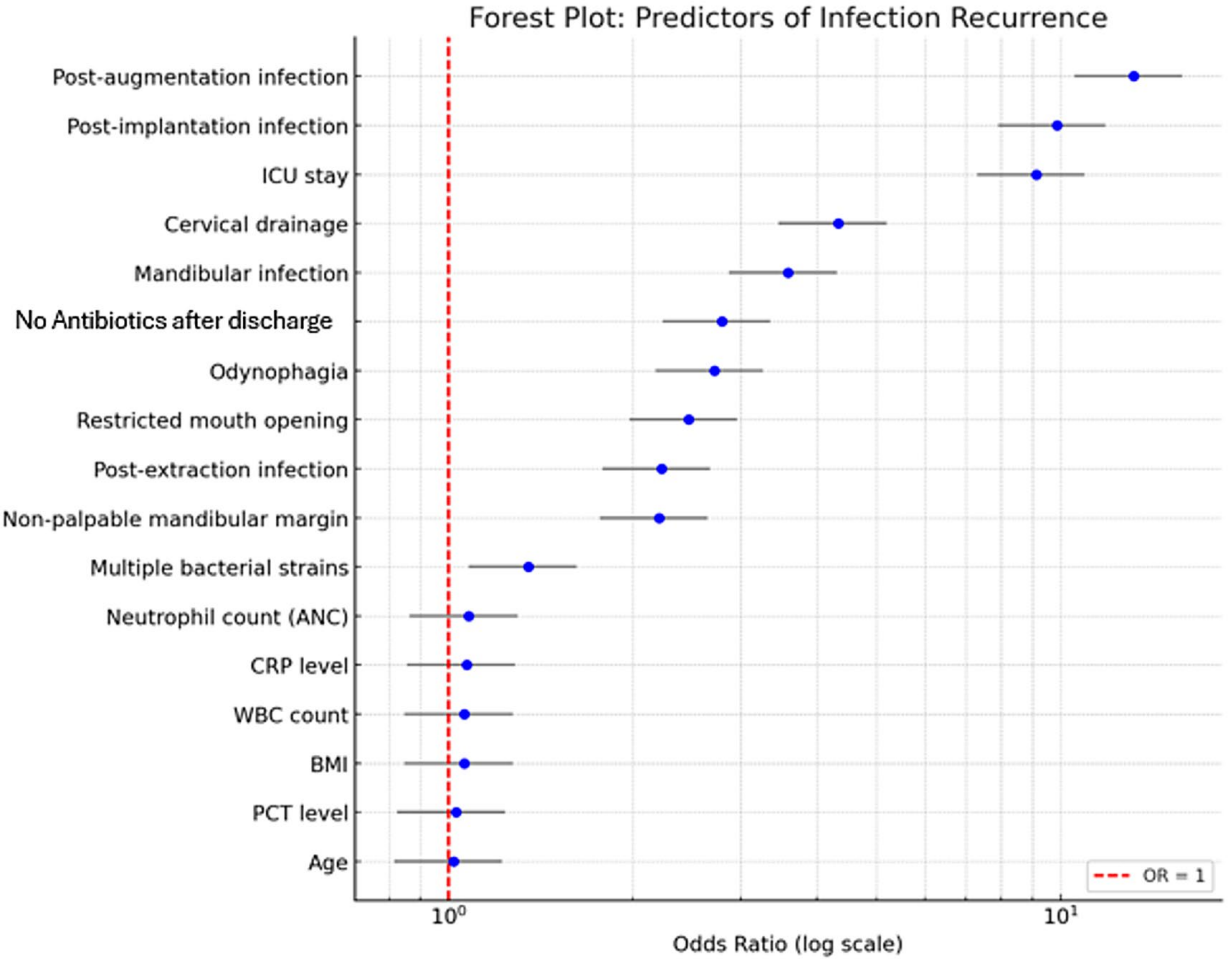
Forest plot summarizing the odds ratios and 95% confidence intervals of significant predictors for infection recurrence

A forest plot summarizing the odds ratios and 95% confidence intervals of significant predictors is shown in Fig. 4.

Detailed point allocations, predictor characteristics, and their clinical interpretation are summarized in Table 7.

**Table 7:** Clinical recurrence risk score. Assigned points were derived from the odds ratios using a simplified proportional weighting method, resulting in a cumulative score ranging from 0 to 31 points. The proposed risk categories are defined as follows: low risk (0–6 points), moderate risk (7–13 points), and high risk (≥ 14 points)

Predictor	OR	Points	Comment
Age	1.02	1	Weak individual predictor
BMI	1.06	1	Additive with age
Mandibular infection	3.59	2	Strong anatomical risk factor
Post-extraction infection	2.23	2	
Post-implantation infection	9.87	3	Highest individual procedure risk
Post-augmentation infection	13.2	3	Highly vulnerable group
Restricted mouth opening	2.47	2	Indicator of disease severity
Odynophagia	2.72	2	Proxy for deep space involvement
Non-palpable mandibular margin	2.21	2	Indicates deep or diffuse swelling
CRP level, mg/dl	1.07	1	Inflammatory burden
WBC count /nL	1.06	1	Standard infection marker
ANC count (%)	1.08	2	Stronger than total WBC
PCT level, ng/ml	1.03	1	Less specific but relevant
Cervical drainage	4.33	2	Reflects advanced infection
Antibiotic therapy after discharge	2.8	2	Suggests postoperative consideration
Multiple bacteria strains	1.35	1	Suggests complex flora
ICU stay	9.13	3	Strongest overall risk indicator

Abbreviations: OR = Odds Ratio; BMI = Body Mass Index; CRP = C-Reactive protein; WBC = White Blood Cells; ANC = Absolut Neutrophil Count; PCT = Procalcitonin; ICU = Intensive Care Unit

## Discussion

This study aimed to assess the incidence of oromaxillofacial infection recurrence after in-hospital treatment and to evaluate the associations between patient- and treatment-related variables, recurrence rate, and hospital length of stay (LOS).

The demographic characteristics of our cohort are consistent with previous studies [20–24]. The recurrence rate of 5.01% observed in our study aligns with the reported range of 3–11% in the literature [2, 21–25]. Liau et al. reported a 4.5% reoperation rate among 672 patients, while Christensen et al. found a higher recurrence rate of 11.2% in a smaller cohort [23, 25]. Differences between studies may result from variations in patient populations, diagnoses, and treatment protocols.

We hypothesized that specific variables would be associated with recurrence and might offer opportunities for outcome improvement. Our multivariate analysis identified key predictors for both recurrence and extended LOS: advanced age, increased BMI, mandibular involvement, infection following extraction or augmentation, clinical symptoms at admission (restricted mouth opening, dysphagia, non-palpable mandibular margin), elevated inflammatory markers (CRP, leukocytes, neutrophils, procalcitonin), and cervical surgical approach.

Older age has been linked to a higher risk of infection recurrence in some studies [23], although other investigations did not find significant associations [22, 24, 26]. In the study by Kaercher et al., reoperation rates were higher in older individuals than in younger people, although the difference was not significant [27]. Possible explanations include reduced immune competence and the presence of comorbidities that impair healing [28–30]. Additionally, elderly patients may face challenges in maintaining postoperative oral hygiene due to reduced mobility.

Increased BMI was significantly associated with both recurrence and LOS in our cohort. Although literature directly addressing this association is scarce, it is plausible that higher adiposity impairs antibiotic distribution, complicates surgical access, and facilitates abscess spread.

Contrary to our initial assumptions, smoking status was not significantly associated with recurrence, in agreement with other studies [22–24, 26].

Mandibular infections posed a significantly higher recurrence risk compared to maxillary infections, increasing the odds of recurrence 3.59-fold. This finding is supported by previous studies reporting a higher complication rate in mandibular infections [24, 31, 32]. The complex anatomy and proximity to multiple fascial spaces may explain the increased surgical difficulty and higher recurrence risk.

Post-extraction and post-augmentation infections were independently associated with higher recurrence rates and increased hospitalization. These findings may reflect tissue trauma, altered bacterial colonization, or postoperative complications following dental interventions.

While no specific abscess location predicted recurrence, infections involving multiple spaces have been associated with increased risk [22–24]. The present study also confirmed that restricted mouth opening, dysphagia, and non-palpable mandibular margins at admission correlated with higher recurrence risk and longer LOS [26, 33]. These clinical signs may reflect advanced disease spread, complicating surgical drainage.

Elevated inflammatory markers (CRP, leukocytes, neutrophils, procalcitonin) were significant predictors of both recurrence and increased hospitalization, corroborating findings from previous studies [3, 23, 24, 26, 34–37]. The magnitude of systemic inflammation may thus serve as a valuable marker for disease severity.

Regarding surgical technique, the use of a cervical approach for drainage was associated with a 4.33-fold higher recurrence risk compared to intraoral access, consistent with previous findings [24, 33]. This may reflect the more extensive spread of infection requiring extraoral drainage.

Interestingly, pre-hospital antibiotic treatment was not significantly associated with recurrence, confirming previous observations [26]. However, patients who received antibiotic therapy after discharge exhibited a higher recurrence risk, possibly reflecting earlier discharge in patients with incomplete infection resolution.

The presence of multiple bacterial strains increased recurrence risk, although specific pathogen types did not. This finding suggests that polymicrobial infections complicate antibiotic selection and treatment effectiveness.

Finally, a postoperative ICU stay was associated with a 9.13-fold increased recurrence risk, highlighting the severe disease burden among these patients.

It is noteworthy that in 61.7% of recurrence cases, the initial infection focus was no longer present at the time of diagnosis. However, persistence of the original focus was not statistically associated with recurrence risk, indicating that recurrence mechanisms are multifactorial and not solely dependent on residual infection.

Our study has several limitations. First, the retrospective design introduces potential biases, including incomplete documentation and variability in clinical management. Second, differences in surgeon experience and inconsistencies in medical record detail may also have influenced the results. The management of the odontogenic source following the initial infection was not evaluated in greater detail due to the retrospective nature of the study and the lack of standardized documentation, particularly for patients referred from external practitioners. As a result, detailed information regarding the timing, extent, and quality of dental/surgical interventions was often incomplete or unavailable. This limits our ability to assess the potential impact of definitive odontogenic treatment on recurrence risk and represents a relevant constraint in the interpretation of our findings. Third, we were unable to provide the duration of follow-up after hospital discharge for the entire study sample. This is because the post-discharge follow-up care was administered by various referring dental colleagues or maxillofacial surgeons. Therefore, a further important limitation is the assumption that patients lost to follow-up did not experience a recurrence. While this approach is commonly used in retrospective analyses, it may have resulted in an underestimation of the actual recurrence rate, particularly if patients with unresolved or recurrent symptoms were less likely to return for follow-up care. This introduces a potential source of attrition bias, as the outcomes of patients not captured in the follow-up period may differ systematically from those who remained under observation. Consequently, the findings—especially regarding recurrence rates and the predictive performance of the risk score—should be interpreted with caution. Furthermore, due to decentralized post-hospital follow-up, data on the post-discharge management of the infection source were unavailable for patients with favorable outcomes. As a result, it was not possible to verify whether definitive treatment was completed in these cases, nor to include them in statistical analyses regarding treatment adequacy and recurrence risk. Fourth, variability in LOS and patient compliance post-discharge may limit generalizability. We acknowledge the limited statistical power in subgroups of certain variables with small sample sizes. Variables with substantial missing data were excluded from the multivariable models to preserve model validity. Fifth, the lack of data on oral hygiene status and treatment compliance with postoperative care and medication regimens may potentially influence the risk of recurrence. These patient-related factors are known to play a critical role in the healing process and in the prevention of recurrent infections, particularly in odontogenic maxillary sinusitis. Their absence from the analysis limits the ability to adjust for potentially important confounders and may have influenced the observed recurrence rate. As such, the interpretation of the identified risk factors should take into account the possibility that unmeasured differences in oral hygiene or treatment adherence may have contributed to the outcomes. Sixth, defining LOS as ≥ 5 days as prolonged was arbitrary and not clinically relevant. Therefore, no statistical analysis was conducted to correlate outcomes with a cut-off value of 5 days of LOS as a binary classification. This is because LOS is influenced not only by the variables examined, but also by physicians’ perspectives and institutional financial policies. Using a random value like 5 days could potentially mislead readers to incorrect take-home messages.

The Recurrence Risk Score developed in this study should be interpreted as a preliminary, exploratory and hypothesis-generating tool derived from retrospective data. While the score integrates statistically significant predictors from multivariate analysis, it lacks external validation. Therefore, prospective external validation in independent patient cohorts is a prerequisite before this score can be considered for routine implementation in clinical practice. Until such validation is achieved, the applicability of the score in routine clinical decision-making remains limited. Furthermore, due to the relatively small number of recurrence events and the associated risk of model overfitting, the derived odds ratios and corresponding score weights should be interpreted with particular caution.

Nonetheless, this large patient cohort provides valuable insights into factors associated with infection recurrence and hospitalization duration. Early identification of high-risk patients based on demographic, clinical, and laboratory findings can improve treatment strategies and outcomes. Future prospective studies are warranted to validate these predictors and the introduced risk model and optimize management protocols and its clinical applicability.

## Conclusion

The results of this study suggest that increased age and BMI, mandibular involvement, post-extraction or post-augmentation infections, clinical symptoms at admission (restricted mouth opening, dysphagia, non-palpable mandibular margin), elevated inflammatory markers, cervical drainage approaches, and stay in ICU are associated with both a higher risk for recurrence and increased hospital stay. Multiple bacterial strains and no continuous antibiotic therapy after discharge also indicated a higher risk of recurrence, while surgery under intubation anesthesia was linked to an increased length of stay only.

These factors should be identified early and incorporated into the clinical decision-making process. However, the developed Recurrence Risk Score represents a preliminary and exploratory tool and requires prospective external validation in independent cohorts before it can be considered for routine clinical application. A structured, evidence- and experience-based treatment strategy—particularly regarding the choice of surgical approach—may help reduce complication rates, improve outcomes, and shorten hospitalization.

## Data Availability

The datasets generated and/or analyzed during this study are available from the corresponding author upon reasonable request.

## Declarations

### Presentation

Preliminary data from this study will be presented at the 4th National Congress of Dental Societies (DGZMK/DGMKG), Berlin, Germany, 30 October–1 November 2025.
